# Hospital Meetings, &c.

**Published:** 1901-03-16

**Authors:** 


### HOSPITAL MEETINGS, &c.

### THE LONDON HOSPITAL.

The quarterly Court of Governors of the London Hospital was held on Wednesday, 6th inst., the Hon. Sidney Holland in the chair. He gave the substance of the report as follows:—The quarter had been the busiest the hospital had ever known. There had been 730 patients in the wards in one day; 686 had been the average number per day. The demands on the hospital were almost more than it could meet; the number of patients during the year had been 12,746. The average residence was 19·81 days, as against 19 in the previous year. It was a very extraordinary thing that 9,000 operations had been performed under anæsthetics—there was nothing like it in the country. Although they had spent the vast sum of £10,000 on the building of the temporary wards and the many numberless arrangements for carrying on the work during the alterations, not a single bed had been closed. They had felt it their duty, there being no hospital in that part of London to relieve them, not to refuse patients during the progress of the alterations. The new isolation ward was nearly finished; the new post-mortem department was in full use; the pathological and out-patients' departments and the alterations in the centre block were being proceeded with. Three thousand two hundred and forty-five children under twelve had been treated during the year, and many more might be added as children if they included those up to sixteen years of age. He did not think many people realised that this was the largest Children's Hospital in the United Kingdom. The Lupus cure was exceedingly successful. It had been started by H.M. Queen Alexandra. The number of patients on the list was so great that they could not reach them for two years. Every case has been greatly benefited, and many had been absolutely cured. There was, of course, no cure for some. Two lights were now in use; these required eight nurses and two sisters, meaning an expenditure of £550 a year in wages alone. There must also be a man to work the electrical part of the appliance. It was of no use disguising the fact that it was a very expensive matter; "but," said Mr. Holland, "we should not wish to draw back, should we?" The Governors, he thought, owed it to the Queen to continue. In reply to a question he said he had been about to add that though in the morning patients were treated entirely free of charge, some paying patients were received in the afternoon. The committee had given directions for the preparation of plans for improving the east and west wings of the hospital. One of the new wards would provide very much larger and better accommodation for the Jews; they had been very liberal themselves in this, Mr. Raphael having given £10,000 for these wards. The sum of £60,000 had been received for the Convalescent Home at Felixstowe, and a contract for £11,000 had been accepted. It was evident that they must make up their minds to spend £370,000, unless they wished the hospital to go backwards instead of forwards. How was this expenditure to be met? The Prince of Wales's Fund had every year given £5,000, on condition that they spent £100,000 of their capital. The sum for which they had to ask the public was £138,000 during the next five years. He did not believe the public would fail in helping them to meet the needs of the poor people of that part of London. He had received during the quarter two very angry letters from governors protesting against pensions being given. He thought they had before them only two alternatives—either to pay very highly for present services, or to give smaller pay and a subsequent pension. With regard to Mr. Rampley, he had been forty years in the hospital, and had been present at 40,000 operations as dispenser. The hospital prided itself on its nursing staff; a great number of nurses had been sent to private patients during the year, and the reports were so good that one would hardly believe there were such good women in the world. Miss Hobbs, so well known as Sister Rachael, had left them to set up a nursing home in Gower Street; another great loss had been the Baroness Van Eck, who had retired for family reasons; several others had obtained appointments elsewhere. The Queen had expressed great satisfaction with the 26 nurses who were chosen for the war; she came to say good bye to them, and to give them useful presents of shawls, &c. She was very pleased that they had stuck to their work and had not wished to return when the excitement was over. One had died, and two were ill with enteric; the others were doing their work well. They had sold out £12,000 of New Zealand stock; and the loan to the Medical College had been increased to enable them to build; this latter was however only an investment, as the money would be returned. The School Board had asked for a certain property in Rutland Street; this, however, was very valuable to the hospital, and he was glad to say the Board had accepted another piece in Romford Street. He hoped the new Borough Council would close Romford Street, which was a dangerous slum; Lord Rowton was building one of his lodging-houses close by. The cost of the new isolation block by St. Philip's Church had been entirely covered by the donations of a gentleman who had already given £22,000 and had now added the sum of £10,000. They might congratulate themselves on the fact that neither for site, building, nor furniture had it cost them a farthing. After reading the list of special donations, which included £900 from the Hospital Saturday Fund, Mr. Holland formally moved the adoption of the report, which was seconded and carried. The Governors were then asked to sanction certain agreements which had already been before the Estates Committee, and the meeting closed with thanks to the chairman.

### CITY OF LONDON HOSPITAL.

The annual meeting of the City of London Hospital for Diseases of the Chest (Victoria Park) was held at Gresham College on Thursday, 7th inst. The chair was taken by Joseph Benson, Esq., L.C.C., chairman of the Committee of Management. About thirty governors were present, including a large number of representatives of working men's societies.

The Secretary read a letter regretting his inability to attend, from Sir Edward Sassoon, owing to recent events in the House of Commons.

The Chairman then made some remarks on the report, which was taken as read. After alluding to the death of Queen Victoria, he said the doctors were of opinion that a longer stay was beneficial to patients, therefore the average number of days for each patient had been 52, as against 47 in the previous year. The average number of patients per day was 116, compared with 103 in the previous year. The average cost per head was £1 10s. 0¾d., *i.e.*, 2s. 1¼d. less than in the previous year, and 5s. 5¼d. less than in 1898; this was notwithstanding the increased price of provisions. Though the finances had suffered on account of the war, there had been a considerable amount of public support; the annual subscriptions had been £16 less than in 1899, and the donations had suffered because the public dinner had been abandoned. The Prince of Wales's Fund took very great care to personally investigate by experts in hospital management before giving its grants; they had increased the grant to £1,600. The hospital had also been exceedingly fortunate in legacies. The increase in expenditure was due to a larger number of beds being occupied, and to the increased prices of provisions; the total expenditure had been £11,165, as against £10,605 in the previous year. The sum received from the working men's societies had been less than last year. One of the most satisfactory signs of progress in the present day was the desire to prevent the spread of consumption; and the question of open-air treatment had come very decidedly to the front during the last few years. They had adopted as far as possible a modified form of this treatment by means of balconies, and he asked for a careful consideration by the Governors of the medical report signed by Drs. V. D. Harris and E. Clifford Beale, in which the details of 294 cases were collected. They included instances of "early," "moderate," and "advanced" disease.

"Of these, 223 cases showed decided improvement after varying periods of treatment, and in many instances the improvement was striking, but taking them altogether it would appear that 75·85 per cent. approximately represents the average of cases improved. By the term "improved" was meant (*a*) gain of weight; (*b*) reduction of temperature; (*c*) diminution of the special symptoms, such as cough and shortness of breath, with improvement of appetite and increase of general bodily vigour. In some cases there was marked improvement of physical signs. Of the 223 patients who exhibited improvement, 209 gained in weight, and of these, 27 each gained a stone or more, 9 gained over 20lbs. each. The total gain of weight amounted to 1,847lbs., giving an average of 8·81bs. per patient. As the average duration of the stay of the patients in the hospital was 7 weeks and 3 days, it would be seen that the average gain per patient per week was more than 1lb. As regards the stage of the disease, the highest percentage of improvement appeared in the early cases, and especially in those in which one lung only was affected. Of these early cases no less than 88 per cent. were recorded as improved. The result of the treatment in the advanced cases showed a surprisingly large percentage, viz., 68 per cent. This would probably be accounted for by the considerable number of cases in which the disease, although advanced, was practically quiescent. Of the intermediate cases, which included cases of disease of both lungs of long standing, the improvement rate appeared to have been 65 per cent. With respect to the ages of the patients, the highest percentage of improvement appeared in those over 40 years of age, viz., 81 per cent. This would probably be accounted for in the same way as had been already suggested for cases of advanced disease. Next to these came the patients up to and including 20 years of age, viz., 78 per cent. Of the patients between 20 and 30, the improvement rate was only a little lower, less than 1 per cent. The period between 30 and 40 would appear to have been the least favourable, but even then it amounted to 70 per cent."

In connection with this report, the chairman gave some statistics for London, and stated that the death rate per annum from consumption was something like 8,500. In Hampstead a man had three times a better chance of life than, *e.g.*, in Finsbury. The evil of overcrowding had a close connection with this state of things; it was an evil that must be dealt with radically. Another matter to which he would draw special notice was the accommodation for nurses; they slept on the same corridors as the patients, and some of their rooms were even underground. They would like to build a Nurses' Home in the grounds—there was ample space—so that the greatest possible change and comfort should be ensured to them when off duty. The matter was being dealt with by the Committee of Management.

Mr. Alexander having seconded, the report was adopted. Dr. Eustace Smith, who retired by rule after thirty years' service on the medical staff, was elected to serve on the Committee of Management, to which two other additions were also made.

Mr. Morton Latham, speaking on a vote of thanks to the chair, laid great emphasis on the need for the nurses' accommodation already referred to. The point to which he wished particularly to refer was that consumption was now being recognised as an infectious disease. It was proposed to include it among notifiable diseases. It was therefore absolutely the duty of the Governors to say that the nurses should not be in the same building with the patients when off duty. It had been said that it would be a very good thing if all buildings used for infectious diseases were built of wood, and burnt down every ten years. He urged the attention of the Governors to the matter on these grounds.

### NEW HOSPITAL FOR WOMEN.

The Annual Meeting of the Governors of the New Hospital for Women, Euston Road, was held on Friday, 8th instant; the Bishop of Islington presided. The report for 1900 showed that the hospital now contained 50 beds; the in-patients had numbered during the year 604; of these 223 were medical cases, 348 surgical, and 33 ophthalmic In the out-patients' department there had been 6,764 new patients, 7,532 renewed, or a total of 33,032 visits. In the ophthalmic department 600 new cases had been seen, and in the recently formed children's department, 228, making 3,253 and 1,397 attendances respectively. 241 cases had been attended in the maternity (out-door) department, with no maternal death. There were 10 operative cases and four sets of twins. Miss Harris, M.B., had resigned her post, and Miss Lucy B. Clapham, M.B., had succeeded her in this department. The finances, which had caused great anxiety last year were now satisfactory; there was a balance in hand, thanks to the energy of the secretary and friends. The Cottage Convalescent Home continued to be greatly appreciated.

The Chairman, in moving the adoption of the report and balance sheet, said he thought the expenditure would compare very favourably with that of other hospitals, since it was proportionately more expensive to carry on a small hospital than a large one. When a student at Guy's Hospital, he had often been grieved that poor women could not have ladies to attend them; and if this hospital existed only for its midwifery department, it would be doing a grand work. The work done here destroyed the idea that women were incapable of the highest achievements of medical skill. Dr. Payne seconded. He thought it was better that a hospital should be a little in debt than to have a balance in hand; it had then more claim on the public. It should be recognised that a hospital did not exist only for the nursing of patients or the education of students; it should also promote the science of medicine. The scientific aspect was by no means neglected here; the pathological arrangements were excellent; he thought, however, that the addition of a clinical laboratory was a project which might commend itself. The report and balance-sheet were adopted.

The Rev. E. F. Russell, in proposing the re-election of the committee, thought that the absence of a children's ward was a serious defect; he hoped the day would come when, in such a ward, all kinds of new discoveries would be made; no more fitting memorial could be raised than this by those who wished to perpetuate the memory of departed friends. Mr. Turle, in seconding, quite agreed that the laboratory should be added to; unfortunately space was limited.

Mrs. Garrett Anderson, in proposing three new vicepresidents, said that but for this hospital, and the opportunities of study and practice which it offered, the medical women of England would never have risen above mediocrity, whereas now the top rank was open to them. Mr. Dryhurst, seconder, said that owing to careful management the average cost per patient was in 1900 £1 12s. 10d., as against £1 14s. 2½d. in the previous year.

Mrs. Scharlieb said that at any rate for India and China medical women needed to be even more highly equipped than their brothers; in those isolated stations it was "sink or swim." This hospital was now looked upon as a sort of mission centre for the propagation of a gospel of physiological righteousness. In no hospital (except the Temperance Hospital) was so little alcohol used per head. The treasurer protested against the idea that debt was a good thing; women liked to pay their way. One or two speakers having said that a telephone was greatly needed, an offer was very generously made by Mr. Turle to provide one, and very heartily accepted by the meeting.

### Royal Westminster Ophthalmic.

The annual general meeting of the Royal Westminster Ophthalmic Hospital was held on March 8th, Sir Charles Turner, chairman of the Committee of Management, presiding. In moving the adoption of the report he said he had recently had occasion to look into the past history of the institution, and one thing that struck him was how thoroughly they deserved the title "Royal." The hospital was established in 1816 under the presidency of the Duke of York, and very shortly afterwards the King, George IV. and all his Royal brothers became patrons. William IV. as King was a patron, and throughout her reign her late Majesty Queen Victoria was so also. The Duke of Wellington was their first president, and no doubt through his influence the King promised them a site. In fulfilment of this promise the piece of ground they now occupied was made over to them in 1831—not freehold, however, but at a very moderate rent, and this rent was for some years paid, half by the War Office and half by the Admiralty, to the Board of Works. The reason of this was that it was intended to be a school of ophthalmic surgery for officers of both services; and throughout the years since that time they had maintained an efficient school. During the past year twenty-two qualified men, in addition to their students had attended for various periods of study. Several of their students and officers had taken part in the great struggle now going on in Africa, and the committee had that day elected as house surgeon a gentleman who had only just returned from the front. During the year their out-patients numbered 10,532, and the in-patients were 689 as compared with 630 in the previous year. Happily the public who supported them had shown increased liberality, the donations having risen from £932 to £1,220. Their subscriptions had, however, fallen off somewhat, the result being a net increase of £340. He regretted to hear the Building Fund had not been supported quite so well as they could have hoped, but that it was thought was due to the want of a definite and fixed policy. If they could make up their minds either to rebuild where they were or to build a new hospital elsewhere they might look with confidence for increased support. As to their present position they were hindered, because their neighbours the Charing Cross Hospital had secured all the available sites. They must, however, soon come to a decision in the matter. The Chairman then referred in suitable terms to the death of the Queen and also to that of the Duke of Wellington and Admiral Somerset, vice-presidents, of Lord Gort and Lord Loch, of Major-General Sim, and other supporters, during the year. He was sure the meeting would sanction the committee's action in presenting an address of condolence to the King and also a request for the continuance as King of the patronage which, as Prince of Wales, he was pleased to accord them.

The accounts showed the ordinary income of the year as £2,754 and the extraordinary income (legacies) as £326. The ordinary outlay was £2,370 and the extraordinary outlay £12 on building improvements and £300 transferred to the building account.

The report and accounts were adopted, and Messrs. Samuel Spencer, Edward Allen, and John Griffith, and Miss Emily Levy having been elected honorary life governors, votes of thanks brought the proceedings to a close.

